# Potential Application of Tea Polyphenols to the Prevention of COVID-19 Infection: Based on the Gut-Lung Axis

**DOI:** 10.3389/fnut.2022.899842

**Published:** 2022-04-14

**Authors:** Lei Xu, Chi-Tang Ho, Yanan Liu, Zufang Wu, Xin Zhang

**Affiliations:** ^1^Department of Food Science and Engineering, Ningbo University, Ningbo, China; ^2^Department of Food Science, Rutgers University, New Brunswick, NJ, United States

**Keywords:** tea polyphenols, COVID-19, gut microbiota, gut-lung axis, antiviral

## Abstract

Coronavirus disease 2019 (COVID-19) disrupts the intestinal micro-ecological balance, and patients often develop the intestinal disease. The gut is the largest immune organ in the human body; intestinal microbes can affect the immune function of the lungs through the gut-lung axis. It has been reported that tea polyphenols (TPs) have antiviral and prebiotic activity. In this review, we discussed TPs reduced lung-related diseases through gut-lung axis by inhibiting dysbiosis. In addition, we also highlighted the preventive and therapeutic effects of TPs on COVID-19 complications, further demonstrating the importance of research on TPs for the prevention and treatment of COVID-19 in humans. Based on this understanding, we recommend using TPs to regulate the gut microbiota to prevent or alleviate COVID-19 through the gut-lung axis.

## Introduction

Human pathogenic coronavirus, including severe acute respiratory syndrome coronavirus (SARS-CoV) and SARS-CoV-2, it binds to the angiotensinogen-converting enzyme 2 (ACE2), a recently discovered mono-carboxypeptidase and the first ACE homolog, and then enters the cell (1). SARS-CoV S1 contains a receptor-binding domain (RBD) that explicitly recognizes ACE2 as its receptor (2), and tea polyphenols (TPs) have been found to bind to RBD to inhibit virus invasion (3). Numerous studies have demonstrated TPs to prevent obesity, diabetes, cardiovascular disease, cancer, and antiviral activity and fight diseases caused by oxidative stress and inflammation (4, 5). For instance, when the balance between the accumulation of reactive oxygen species (ROS) and the body's antioxidant process is disturbed, oxidative stress can be induced, causing damage to cells and tissues, thus leading to various diseases (6). However, when TPs enter the body, the activity of antioxidant enzymes increases, the inhibition of lipid peroxidation, and the production of ROS in the body can be promoted to achieve the antioxidant effect (7). These effects are also likely to help alleviate a range of complications caused by the new coronavirus.

Tea is the most popular beverage besides water and the most widely used (8). In China, tea consumption has been more than 5,000 years. TPs are a mixture of phenolic compounds extracted from tea leaves. In terms of concentration, tea catechins are one of the most important bioactive substances in tea leaves, accounting for 60–80% of total polyphenols. Catechins are the main polyphenol compounds in tea, including epigallocatechin-3-gallate (EGCG), epigallocatechin-3-gallate, epicatechin-3-gallate and epicatechin, the content and activity of EGCG was the highest (9).

It is known that the dysbiosis of the human gut microbiota is associated with various health conditions, including respiratory tract infections (RTI) via the gut-lung axis. The gut microbiota is involved in various physiological responses, including nutrient absorption, energy regulation, glucose metabolism, and immune system regulation (10). Perhaps only 1.9% of the gut microbiome is heritable, while more than 20% of the biodiversity of the microbiome is derived from the environment (including diet). TPs can effectively modulate gut microbiota composition, thereby effectively improving gut microbiome and host health. For many, COVID-19 brings few symptoms, but others are life-threatening due to SARS-CoV-2. While certain gut microbes have been linked to adverse outcomes from viral infections, some researchers suggest using these bacteria as biomarkers. If gut health affects the prognosis of COVID-19, we should use it to better control and prevent COVID-19. TPs have been used in research in the fields of immunity, psychiatric diseases, cardiovascular and metabolic diseases, and have achieved certain achievements. It can be seen that it is reasonable to use tea polyphenols to regulate intestinal microecology and prevent and intervene in COVID-19 (11). Therefore, improving the nutritional status of patients and enhancing the body's immunity by regulating the microbiota is of great significance for the treatment of novel coronavirus pneumonia. In this review, we summarized the possible use of TPs to prevent viral infections. In addition, the mechanism of action of TPs against COVID-19 was discussed from the perspective of the gut-lung axis.

## The Important Role of Gut Microbes in Covid-19

Patients with COVID-19 show signs of intestinal flora imbalance, which can cause intestinal damage and damage to the lung and vital organ systems in the event of a pathogenic SARS-CoV-2 infection. Therefore, it is essential to maintain a healthy gut microbiome to optimize the immune system in order to prevent excessive inflammation (12).

### Intestinal Flora and the Gut-Lung Axis

Intestinal microorganisms can interact with the immune system, and the immune cells generated by the immune function between the intestine and the lungs move through the lymphatic system and blood circulation. The interaction network between the intestine and lung tissue mediated by microorganisms and immune cells is called the “gut-lung axis” (13). The imbalance of intestinal flora interacts with lung diseases and respiratory infections. When a deadly influenza virus invades, intestinal flora such as endogenous *Bifidobacteria* will increase, enhancing the host's resistance to influenza (14). The main bacterial phyla of the lungs are the same as the intestines, mainly *Firmicutes* and *Bacteroides*, followed by *Proteobacteria* and *Actinomycetes*, the interaction between the lung microbiota and immunity is also a two-way process (15). “Gut-lung axis” refers to the intestinal flora that can affect and regulate the immunity and function of the lungs, and may be related to acute and chronic lung diseases (16). And patients with chronic gastrointestinal inflammation and other diseases have a higher prevalence of lung diseases. Respiratory influenza infection can cause intestinal injury when lung injury occurs, and influenza infection changes the composition of intestinal microflora (17).

Patients with respiratory infections usually have intestinal dysfunction, and some COVID-19 patients experience gastrointestinal (GI) symptoms, including diarrhea and vomiting (18). The proportion of 651 COVID-19 patients with gastrointestinal symptoms was 11.4%; trends in fever and severity (severe/critical, mechanical ventilation, and ICU admission rates) were significantly higher in COVID-19 patients with gastrointestinal symptoms (19). Experiments in mice have shown that depletion or loss of the intestinal microbiota can lead to impaired immune response and worsen the prognosis of bacterial or viral respiratory infections (20). The gut-lung axis results from complex interactions between microbial components in the gut and lung flora and local and long-term immunity. Mice infected with the H1N1 flu in the nose developed lung infections, a marked change in the composition of the intestinal flora, and an increase in *Bacteroides* (21). Using mouse models of respiratory tract influenza infection found that respiratory tract influenza infection can cause intestinal damage and change the composition of the intestinal microbiome with the increase of *Enterobacteriaceae* bacteria and the decrease of *Lactobacillus* and *Lactococcus* (22). In a meta-analysis, the gut microbiota of 30 COVID-19 subjects, 24 H1N1 patients, and 30 healthy controls were evaluated. It was found that the intestinal bacterial diversity of subjects infected with SARS-CoV-2 was significantly reduced, and the relative abundance of beneficial microorganisms, such as *Bifidobacterium* was also reduced (23). Therefore, it is speculated that SARS-CoV-2 may indirectly affect the intestinal flora related to the intestine-pulmonary axis and damage human immunity, and it can prevent and treat lung infections caused by SARS-CoV-2 by regulating the relevant intestinal flora.

### Changes in the Intestinal Flora of COVID-19 Patients

The intestinal microflora is closely associated with respiratory viral infections and causes various infections through the gut-lung axis (24). In addition, influenza infection will affect the composition of the intestinal flora, and the disorder of the intestinal flora will reduce the host's antiviral immune response, thereby aggravating the lung damage caused by these infections (25). Among them, changes in the intestinal environment and immune factors caused by actinomycetes may aggravate the damage caused by inflammatory bowel disease. Compared with healthy individuals, the fecal microbiome of COVID-19 patients has significantly changed. The baseline abundance of *Coprobacillus, Clostridium ramosum*, and *Clostridium hatheway* correlates with the severity of COVID-19; the abundance of *Faecalibacterium prausnitzii* (an anti-inflammatory bacteria) the degree is negatively correlated with the severity of the disease (26). Sequenced 274 feces samples (including feces from 100 COVID-19 patients) and found that members of the *Bacteroidetes* phylum in patients with COVID-19 were relatively abundant, and the compositional differences in the gut microbiota of COVID-19 were mainly caused by the enrichment of *Ruminococcus gnavus, Ruminococcus torques* and *Bacteroides dorei*, and the depletion of *Bifidobacterium adolescentis, Faecalibacterium prausnitzii* and *Eubacterium rectale* (27). In conclusion, the gut microbiota of SARS-CoV-2-infected patients is altered by a decrease in commensal microorganisms, a loss of bacterial diversity, and an increase in opportunistic pathogens.

Patients with metabolic and GI are considered to be at moderate to high risk of SARS-CoV-2 infection, suggesting that gut dysbiosis directly affects the severity of COVID-19 (28). RNA metagenomics sequencing was performed on the continuous fecal virus extracts of 15 COVID-19 hospitalized patients. Feces with high SARS-CoV-2 infectivity have higher microbiome functions, and demonstrated the increased relative abundance of *Collinsella aerofaciens, C. tanakaei, Morganella morganii*, and *Streptococcus infants* (29). Based on COVID-19 patient data, a blood proteomics risk score was constructed, and it was found that gut microbial characteristics can highly predict the susceptibility and severity of COVID-19 (30). Therefore, intestinal microbial characteristics and related metabolites can be used as potential prevention/treatment targets for intervention, especially for those who are susceptible to SARS-CoV-2 infection.

### Gut Flora Regulates Immunity Through the Gut-Lung Axis

The gastrointestinal tract hosts a complex and highly diverse microbial ecosystem that interacts with the host to ensure the establishment and persistence of immune homeostasis (31). These complex microbial communities provide important genomic and enzymatic capabilities and play critical roles in the immune system's induction, development, and function, protection from pathogens and sustained tolerance to innocuous antigens, and protection of the ecology of the microbiota (32). The gut microbiome is the protective agent during pneumococcal pneumonia, and the gut microbiome enhances primary alveolar macrophage function (20). In an acute lung infection model, oral administration of segmented filamentous bacteria stimulates pulmonary T helper cell responses and reduces *S. pneumoniae* infection and mortality (33). Studies have shown that patients with COVID-19 have lower levels of probiotics (such as *Lactobacillus* and *Bifidobacterium*) (34). Because of the critical role of the intestinal flora and its metabolites in regulating the host's immune and inflammatory response, the regulation of the intestinal flora can be used to prevent and treat COVID-19 and related diseases (such as viral and/or bacterial pneumonia, acute respiratory infections, or flu) has attracted considerable attention.

**Figure 1:**
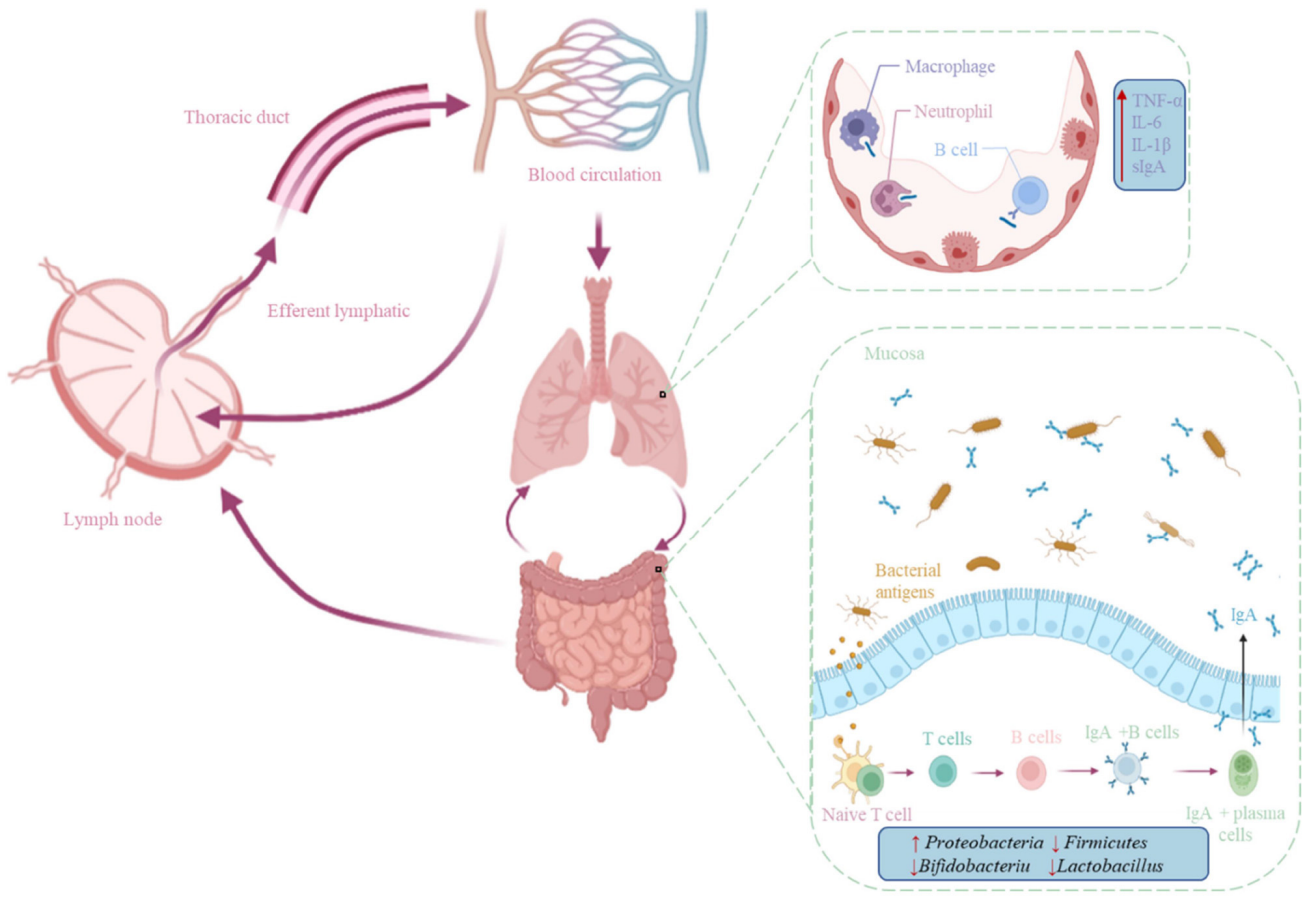
Mechanisms of the gut-lung axis in the immune system. The previous crosstalk between the gut and lung mucosa is through the mesenteric lymphatic system and the lymph nodes in the lungs, and immune cells generated by the immune function between the gut and the lungs move through the lymphatic system and blood circulation.

The interaction between the gastrointestinal tract and the respiratory tract is achieved through a common mucosal immunity. There is persistent crosstalk between the intestine and the pulmonary mucosa through the mesenteric lymphatic system and the pulmonary lymph nodes (35). Short chain fatty acids (SCFAs) induce the expression of dendritic cell and macrophage pattern recognition receptors and regulate cytokine secretion and antibody synthesis (sIgA and IgM) (36). Dysbiosis in the lung affects the immune system and decreased immune cell recruitment leads to increased viral load in the lungs and reduced IFN-α and -β production, which negatively affects T cell priming (37). Germ-free mice display defects in several specific immune cell populations, such as impaired innate lymphocyte function, lack of IgA-producing plasma cells, and, more generally, increased susceptibility to infection (38). Using each of 53 separate bacterial species to single-colonize mice, it was found that the diversity of microbes in the gut ensures the ability of the microbiota to produce consistent immune regulation (39). When circumventing responses to pathogenic infections such as coronaviruses, a healthy gut microbiome may be vital to maintain an optimal immune system, preventing a cascade of excessive immune responses that ultimately damage the lungs and vital organ systems (Figure 1).

## Potential Applications of TPS to Alleviate Covid-19

The gut-lung axis plays an important role in SARS-CoV-2 infection, so targeting the gut-lung axis to treat COVI-19 is particularly important. TPs are considered to be multifunctional bioactive molecules, which have antiviral effects in addition to antibacterial and intestinal flora regulation to enhance immune function (40). Therefore, TPs are considered to have potential preventive and therapeutic effects on COVID-19.

### Antibacterial Effect of TPs

In addition to various pharmacological effects such as antioxidant, lowering blood sugar, and immune regulation, TPs also have potent antibacterial effects, especially for Gram-positive and Gram-negative bacteria (41). Although the current research results on the antibacterial mechanism of TPs are not very clear, researchers generally believe that the mechanism involves many aspects, such as destroying the cell wall membrane structure, interfering with cell growth and division, and inducing oxidative stress (42). Catechins can inhibit bacterial toxins directly by binding to bacterial toxins or indirectly by preventing bacterial toxin secretion or promoting bacterial protease breakdown (43).

The influence of the catechin structure in TPs on the antibacterial effect mainly includes: (1) The complexation of the ortho-phenolic hydroxyl group with the metal ion. The metal ions in bacteria are partly prosthetic groups of enzymes and partly essential bacteria elements. Multiple ortho-phenolic hydroxyl groups of TPs molecules can be used as multi-base ligands to undergo complex reactions with iron, calcium, and other ions to produce precipitation. Induce oxidative stress while depriving bacteria of essential nutrients, thereby affecting bacterial activity, growth, and reproduction (44, 45); (2) The phenolic hydroxyl group and benzene ring structure are combined with proteins. The phenolic hydroxyl group and benzene ring structure of TPs can be combined with bacterial proteins through hydrogen bonds or hydrophobicity, affecting the physiological functions of proteins, thereby inhibiting bacterial infection and metabolic activity (46, 47); (3) The effect of polymerization degree on the bacteriostatic effect of catechins. For example, compared with catechin monomers, its oligomers have higher antibacterial properties, which may be attributed to the polymers having more phenolic hydroxyl groups and benzene ring structures and having a more substantial binding ability with proteins (48). However, compared with catechin oligomers, catechin polymers and polymers have weaker bacteriostatic effects, which may be because the molecular weight of catechin polymers increases with the increase of the degree of polymerization, making it challenging to penetrate bacterial cell membranes (49). In addition, affected by the steric hindrance effect of macromolecules, the activity of the phenolic hydroxyl group is weakened, resulting in a decrease in the antibacterial ability.

### Regulation of Gut Microbes by TPs

TPs can promote the growth of beneficial bacteria in the intestinal tract and inhibit the growth of pathogenic microorganisms in the intestinal tract from regulating the composition of intestinal flora (50). Intestinal microbes are an essential component of the intestinal environment. Intestinal microbes can enhance the function of the intestinal barrier by interacting with the body's metabolism to produce various metabolites and promote mucosal immune homeostasis (51). The intestinal mucosal barrier is a defense system against external infections and self-maintenance and plays an essential role in maintaining intestinal homeostasis and body health. Zhang et al. (52) studied TPs' therapeutic and preventive effects on ileal injury and intestinal flora disorder. The results showed that TPs could reduce inflammatory and oxidative stress markers, increase the levels of antioxidant enzymes and tight junction proteins, effectively improve the intestinal flora imbalance, reduce the damage to the intestinal mucosa and boost the body's immunity.

Ten volunteers who did not drink green tea drank it for 10 consecutive days, and the proportion of *Bifidobacteria* showed an overall increasing trend (53). A mouse model was established to explore the regulatory effect of TPs on the intestinal flora. The study found that after feeding green TPs, specific bacterial communities such as *Bacteroidetes* and *Proteobacteria* still increased, and *Firmicutes* showed a decreasing trend (54). This result indicates that TPs in green tea can improve the diversity of intestinal flora and regulate the composition of flora, thereby improving and maintaining the ecological balance of intestinal flora, which is beneficial to human health. The addition of TPs to calf feed reduces *Clostridium perfringens* in the gut, which is associated with a lower incidence of digestive and respiratory diseases (55). Green tea consumption decreased relative abundance at the phylum level of *Bacteroidetes*. In addition, SCFAs-producing bacteria, including *Faecalibacterium, Coprococcus*, and *Bifidobacterium longum*, increased, while species from *Prevotella* decreased. And SCFAs are important factors regulating cytokine secretion and antibody synthesis (56). In conclusion, the protective effect of TPs on intestinal microflora has been supported by a large number of experimental results. Therefore, the reconstruction of immune homeostasis through the normalization of the intestinal microbiome is considered an effective method to treat COVID-19.

### Inhibitory Mechanism of TPs on SARS-CoV-2

Recent studies have demonstrated that TPs, particularly EGCG, inhibit coronavirus enzymes as well as coronavirus replication *in vitro* (57). However, laboratory and clinical studies have been performed to study the efficacy of green tea consumption in COVID-19 treatment, and the results are promising. SARS-CoV-2 has a high affinity for ACE2, which acts as a receptor for the spike glycoprotein on the surface of coronaviruses to facilitate virus entry. Nrf2 is a cytoprotective transcription factor that regulates the expression of a wide range of genes involved in detoxification, inflammatory, immune and antiviral responses (58). EGCG, *via* activating Nrf2, can suppress ACE2 (a cellular receptor for SARS-CoV-2) and TMPRSS2 (the cell entry that mediates the virus) (59). 3CL protease is required for the maturation of SARS-CoV-2, and numerous experiments have demonstrated that TPs (EGCG and theaflavins) have inhibitory effects on SARS-CoV-2 3CL protease (60). Mice with COVID-19 had lower levels of coronavirus RNA in their lungs when fed EGCG and TPs containing more than 60% catechins (61). Results demonstrated that EGCG treatment decreases viral RNA and viral protein production in the media, therefore, EGCG can inhibit coronavirus replication.

Data from docking simulations and *in vitro* assays suggest that EGCG is capable of inhibiting the SARS-Cov-2 major protease activity and thus can be used to interfere with SARS-Cov-2 infection (62). In a recent study reviewing the antiviral activities of EGCG and theaflavins, the authors suggest that both polyphenols are able to interact with receptors present in the structure of the SARS-CoV-2 virus, thereby inhibiting its replication. In particular, theaflavin-3,3'-digallate (TF3) can be employed as prophylactic agents due to their capacity to bind spike RBD the main binding domain of the S protein located on the S1 subunit of the SARS-CoV-2 virus; and TF3 can directly bind to viral M protease and ACE2 receptors, helping to fight SARS-CoV-2. EGCG can be used as a potential preventive agent because of its ability to dock various active sites of the SARS-CoV-2 virus (63).

Evidence suggests that patients infected with RNA viruses are in a chronic oxidative stress state, which is induced by the activation of phagocytes to produce and release ROS, and leads to the depletion of antioxidant defense systems (64). The increase in reactive oxygen species and the loss of antioxidant defense mechanisms increase the incidence of SARS-CoV-2 infection and the risk of immune dysfunction and death (65). Catechins activate antioxidant enzymes, and the antioxidant power of human plasma increases with the continued intake of green tea. These antioxidant defense systems also protect against oxidative damage in the brain, long-term intake of green tea catechins may be important because cells are often exposed to oxidative stress (66). TPs inhibits certain enzymes involved in reactive oxygen species ROS production by upregulating other endogenous antioxidant enzymes (such as glutathione peroxidase, superoxide dismutase and catalase); while promoting heme oxygenation enzyme 1 expression to reduce ROS production (67). Therefore, it is necessary to supplement dietary antioxidants to improve immunity when managing COVID-19. The use of exogenous antioxidants such as TPs can significantly influence the clinical outcome of COVID-19 by improving patients' health, speeding up the immune process, and thus shortening hospital stay.

**Figure 2:**
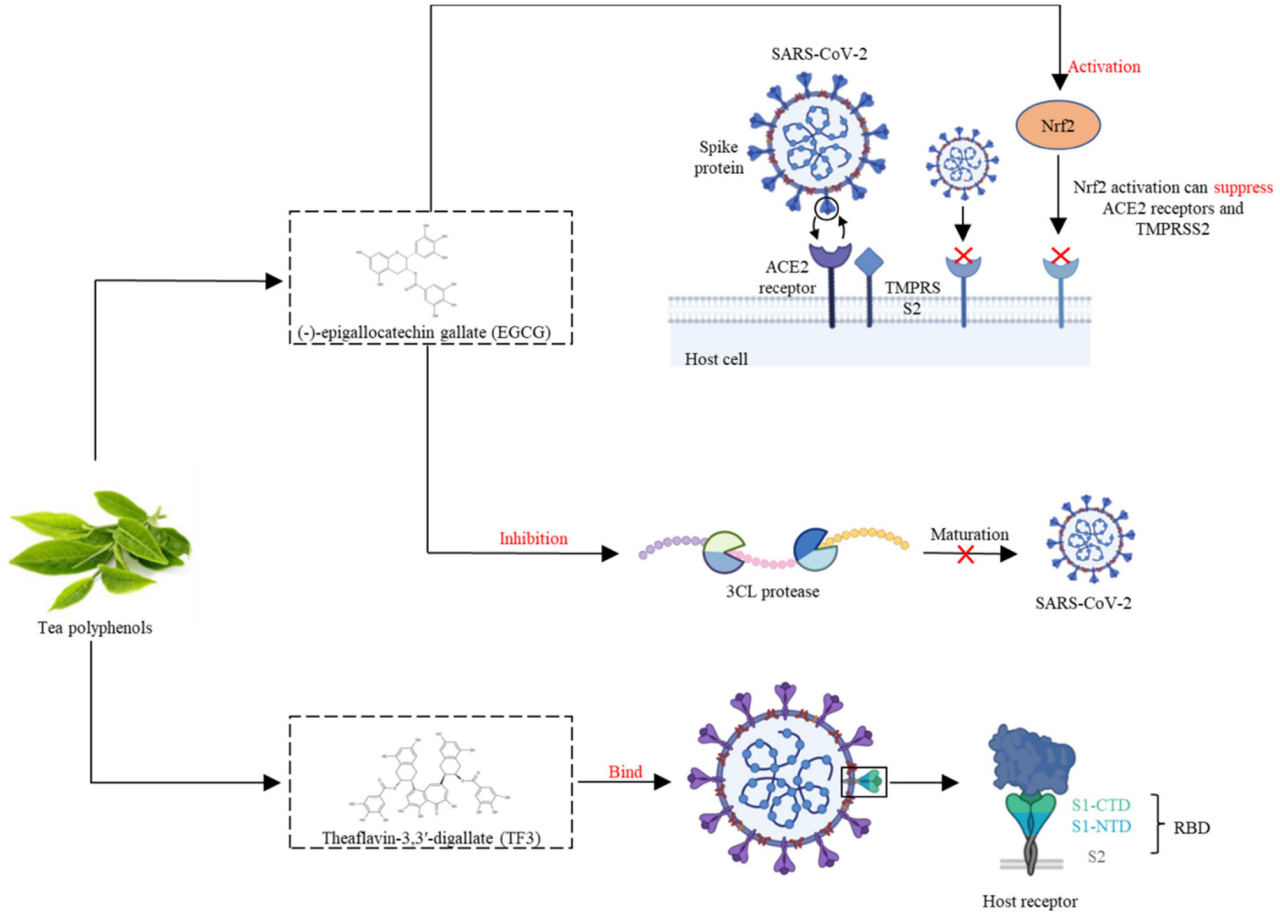
EGCG, *via* activating Nrf2, can suppress ACE2 receptors and TMPRSS2 during SARS-CoV-2 infection. TF3 can bind the SARS-CoV-2 spike receptor-binding domain, and help to fight SARS-CoV-2.

Overall, TPs have antiviral solid and antioxidation properties that may help reduce the risk of developing severe COVID-19 symptoms; these findings highlight the potential for TPs to prevent and treat COVID-19 (Figure 2).

## Reduction of Covid-19 Comorbidity Risk by TPS

In a retrospective study of 1,591 severely ill patients with COVID-19, hypertension was the most common comorbidity (49%), followed by cardiovascular disease (21%), hypercholesterolemia (18%), and diabetes (17%) (68). Patients with COVID-19 can develop complications of lung disease (cough, decreased lung diffusivity, sleep apnea, and pulmonary fibrosis), cardiovascular disease (diabetes, arrhythmia, and myocarditis), and neurological disorder (depression, anxiety, and attention disorders) (69). TP can effectively prevent and treat some complications.

### Pulmonary Fibrosis

COVID-19 patients may have the sequelae of pulmonary fibrosis, with symptoms such as dry cough, fatigue, and dyspnea, leading to weight loss, worsening physical condition, long-term disability, and affecting the patient's quality of life (70). EGCG strongly inhibited neutrophil, inhibited reactive oxygen species activity and inhibited apoptosis of activated neutrophil, enhanced the regression of pulmonary inflammation model, and significantly reduced subsequent fibrosis (71). EGCG reduces NF-κB, TNF-α, and IL-1β, and this blockade may be critical for the upregulation of proinflammatory and fibrotic cytokine genes in models of pulmonary fibrosis (72). But so far, there are no clear and reliable data on the frequency and severity of pulmonary fibrosis in COVID-19 patients.

During treatment of pulmonary fibrotic rats with EGCG, the rats exhibited reduced inflammation, alveolar damage, and vascular congestion, which were associated with the membrane-stabilizing and antioxidant properties of EGCG, demonstrating that EGCG can act as a potential anti-fibrotic drug (73). Oral administration of green tea extract (equivalent to EGCG doses of 300–400 mg/kg) in drinking water to mice almost wholly prevented interstitial and peribronchial fibrosis, >99% reduction in interstitial and peribronchial fibrosis and ~50% reduction in perivascular fibrosis (74). After EGCG (600 mg given orally for 14 days) treatment in 20 patients with pulmonary fibrosis, reverses profibrotic biomarkers in their diagnostic biopsies and serum samples, EGCG treatment was associated with a reduction in fibrogenesis (75). These inhibitory activities of EGCG in rodent models and humans suggest that EGCG may be beneficial for preventing and treating pulmonary fibrosis in COVID-19 patients.

### Diabetes

High-risk patients with severe COVID-19 or death have a variety of characteristics, including advanced age and masculinity, as well as potential health problems such as cardiovascular disease, obesity, and diabetes (76). Preliminary studies have found that diabetes increases the risk of infection with SARS-CoV-2 and increases the severity of COVID-19 (77). In human monocytes, an increase in glucose levels leads to an increase in SARS-COV-2 replication, which is maintained by glycolysis through the production of mitochondrial reactive oxygen species and activation of hypoxia-inducible factor 1α (78). Therefore, high blood sugar may support virus proliferation. Patients with diabetes usually have higher levels of SARS-CoV-2 infection than patients without diabetes. ACE2 knockout mice are more susceptible to high-fat diet-induced pancreatic beta-cell dysfunction than wild-type mice (79); and SARS-CoV infection can lead to hyperglycemia in people with no history of diabetes (80). This finding suggests that coronaviruses might specifically damage islets, potentially leading to hyperglycemia.

Drinking 3–4 cups of tea per day (600–900 mg/day) is often considered to prevent personal obesity metabolic syndrome or reduce disease risk (81). In mice fed a high-fat (60% calorie) diet; we found that EGCG (0.32% of the diet) significantly reduced weight gain, body fat, and visceral fat at 16 weeks (82). A retrospective study of 17,413 Japanese adults aged 40–65 showed compared with people who drank <1 cup of green tea a week, drinking more than six cups a day reduced the risk of diabetes by 33% (83). TPs in reducing plasma cholesterol levels, prevention of hypertension and improving endothelial function in the role of helping to prevent cardiovascular diseases. Weight loss and improved metabolic health may help better cope with COVID-19, whether regular drinking tea (and the required amount) can reduce the risk of COVID-19 infection and related syndromes requires a large number of experiments to prove.

### Depression

In addition to posing a significant threat to physical health, the COVID-19 pandemic also poses a threat to the population's mental health due to increased fear and uncertainty; and disruption to social and economic systems. The prevalence of depression in the general population during the COVID-19 outbreak is 25% (84). Alterations in the composition of the gut microbiota can increase the permeability of the gut barrier, activate systemic inflammatory and immune responses, modulate the release and efficacy of monoamine neurotransmitters, alter the activity of the hypothalamic-pituitary-adrenal axis and function, and alters the abundance of brain-derived neurotrophic factor (BDNF) (85). A deficiency of BDNF may lead to neuroplasticity impairment and depression. The mechanism of the anti-depression effect of TPs is related to the inhibition of HPA axis hyperactivity by reducing serum corticosterone and ACTH levels (86). TPs also have an anti-anxiety effect (similar to anti-anxiety drugs) at lower doses.

**Figure 3:**
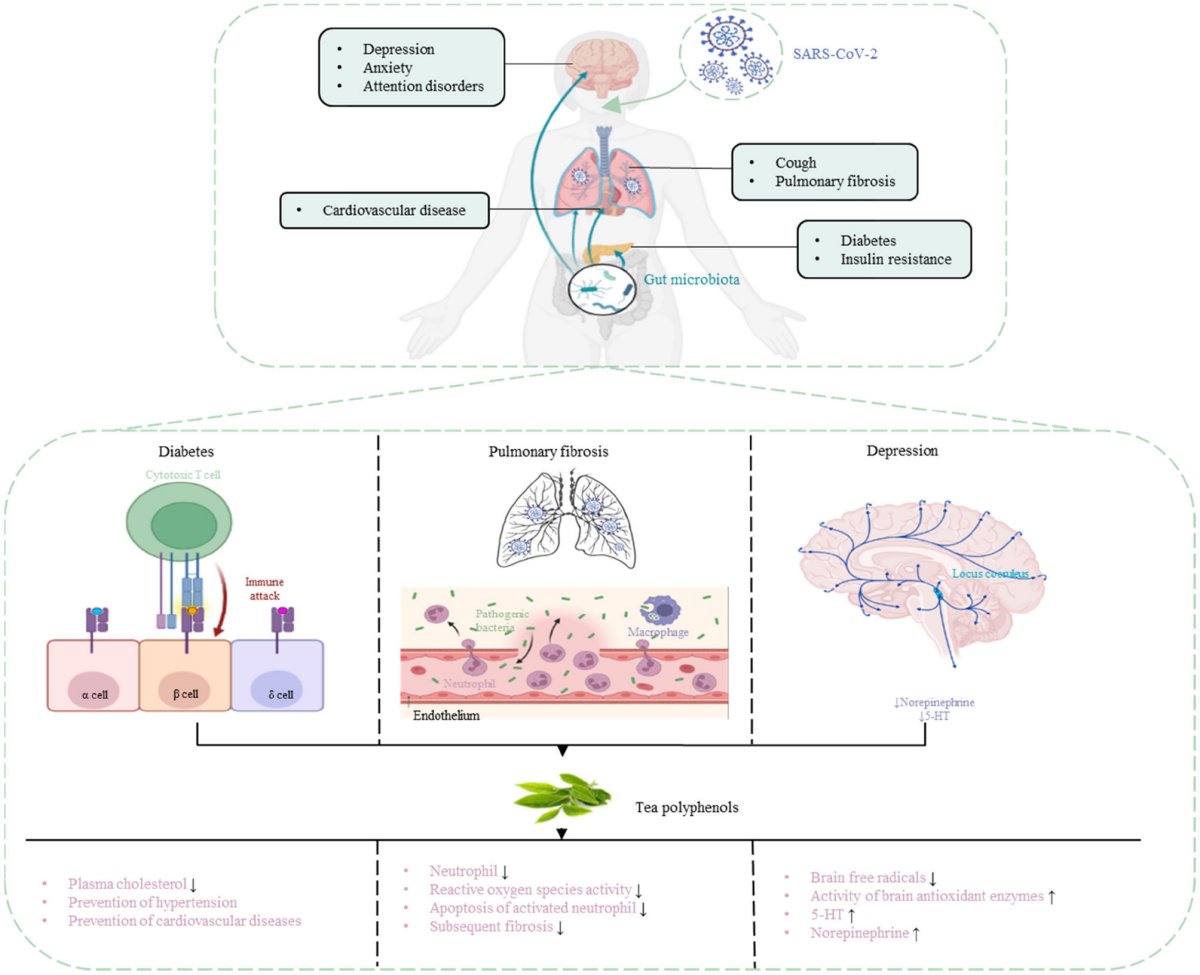
Prevention and treatment of COVID-19 complications with TPs.

An investigation involving 2,011 Finnish general individuals found that daily consumption of tea was negatively correlated with the risk of depression (87). In addition, participants who drank ≥4 cups of green tea per day had a 51% lower prevalence of depressive symptoms compared to those who drank ≤ 1 cup of green tea per day (88). The antidepressant mechanism of TPs may be related to scavenging brain free radicals, regulating monoamine neurotransmitters in the brain tissue of depressed animals, and increasing the activity of brain antioxidant enzymes. Via establishing a mouse model of depression, it was found that the content of 5-HT and norepinephrine in the brain tissue of normal mice was significantly higher than that of depressed mice (89). After TPs were given, the content of 5-HT and norepinephrine in the brain tissue was significantly higher than that in the original depression mice (90). The antidepressant effect of TPs has been proven, prompt prevention of mental health status is also necessary for COVID-19 patients, whether regular drinking tea can reduce the risk of COVID-19 infection and related syndromes needs to be further investigated (Table 1, Figure 3).

**Table 1:** The reduction of COVID-19 comorbidity risk by TPs.

**Comorbidity**	**Risk**	**Experimental model**	**Results**	**Reference**
Pulmonary fibrosis	33%	EGCG doses of 300–400 mg/kg	>99% reduction in interstitial and peribronchial fibrosis and ~50% reduction in perivascular fibrosis	(74)
Diabetes	17%	Drink ≥ 6 cups a day	33% lower risk of diabetes	(83)
Depression	25%	Drink ≥ 4 cups a day	51% lower prevalence of depressive symptoms	(88)

## Dosage and Instructions for Correct Use of TPS

Tea is rich in polyphenols, inexpensive, readily available, and most importantly, safe for long-term use regardless of the patient's age (91). However, it is essential to note that taking EGCG containing preparations during pregnancy may increase the risk of fatal leukemia (92). According to the review of toxicological evidence, the liver is the target organ, and hepatotoxicity is the critical effect, strongly associated with certain dosing conditions (such as mode of administration, fasting) and positively correlated with catechin and EGCG content (93). In the case of oral therapy, changes in hepatotoxicity and serum lipid profiles were evident only at the highest dose of 108 mg/kg/p.o. However, EGCG treatment to achieve appreciable plasma concentrations may concomitantly increase serum lipids, thereby increasing the severity of the liver injury (94). At present, we have not accurately explained the association mechanism between EGCG, liver, and blood lipids, which must be clarified further. Regular consumption of green tea appears to be safe, but high doses of green tea extract in dietary supplements may affect drug metabolism and efficacy (95). Because these products contain concentrated bioactive agents, the doses consumed extensively exceed the doses available from food. Compared to the control group (about 10 mg per day), short-term (3 days) overconsumption of green tea catechins (about 8 grams per day) resulted in a significant increase in liver enzyme activity (by 35–80%) (96). Interestingly, short-term overdose of green tea extract has a more significant effect on drug biotransformation enzymes than long-term small doses. There is no clear information on how much catechins should be taken to achieve the best results. Therefore, it is necessary to evaluate the specific amount of catechin and its possible adverse effects.

According to the United States Department of Agriculture (USDA), the average total catechin and EGCG per 100 mL of brewed green tea was 126.6 mg and 77.8 mg, respectively, based on 1 g tea leaf 100 mL infusion (93). Therefore, every 240 mL serving of brewed green tea provides about 304 mg of total catechin and 187 mg of EGCG. Thirty-six healthy male volunteers took 800 mg of EGCG orally for 10 days and were tested for safety, tolerability, and plasma kinetic behavior; the researchers found that the dose was safe and well-tolerated (97). For adults with normal liver function, the safe intakes limit of 338 mg of EGCG per day in solid form (under-eating or fasting conditions) may be considered. The observed safe level of EGCG equivalent dose (ingestion or fasting) for green tea preparations consumed in beverage form is 704 mg/day (93). In a randomized, double-blind trial of 200 healthcare workers, six capsules per day (including 378 mg of catechin and 270 mg of EGCG) for 5 months were better at preventing the flu virus than a placebo (98).

Numerous experiments are still needed to confirm the specific drug administration (green tea beverage, powdered green tea extract, catechin mixture, catechin alone), dose regimen (different doses, different duration of treatment), and administration pathway management (oral in diet, oral in a beverage) before determining the use of TPs for the treatment of COVID-19.

## Summary and Future Direction

Many experiments have confirmed the safety of tea, and an appropriate amount of TPs will not cause harm to the human body. EGCG is one of the most important catechins in tea, enhancing the body's antiviral ability and gradually being regarded as a potential therapeutic agent for novel coronavirus infection. Although numerous epidemiological and clinical studies have shown that TPs have preventive and therapeutic effects on COVID-19, we lack specific dosages of TPs as dietary supplements or nutraceuticals for the prevention and treatment of COVID-19. To obtain more specific information, well-designed extensive cohort studies and human intervention trials are necessary.

## Author Contributions

LX: conceptualization, validation, and writing–original draft. C-TH: supervision. YL: validation and writing–original draft. ZW: editing. XZ: supervision, writing–review, and editing. All authors contributed to the article and approved the submitted version.

## Funding

This work was sponsored by Zhejiang Provincial Key Research and Development Program (2020C02037) and the Ningbo Natural Science Foundation (2021J107).

## Conflict of Interest

The authors declare that the research was conducted in the absence of any commercial or financial relationships that could be construed as a potential conflict of interest.

## Publisher's Note

All claims expressed in this article are solely those of the authors and do not necessarily represent those of their affiliated organizations, or those of the publisher, the editors and the reviewers. Any product that may be evaluated in this article, or claim that may be made by its manufacturer, is not guaranteed or endorsed by the publisher.
